# Extra-Synaptic GABA_A_ Receptor Potentiation and Neurosteroid-Induced Learning Deficits Are Inhibited by GR3027, a GABA_A_ Modulating Steroid Antagonist

**DOI:** 10.3390/biom13101496

**Published:** 2023-10-09

**Authors:** Sara K. S. Bengtsson, Jessica Sjöstedt, Evgenya Malinina, Roshni Das, Magnus Doverskog, Maja Johansson, David Haage, Torbjörn Bäckström

**Affiliations:** 1 Umeå Neurosteroid Research Center, Department of Clinical Sciences, Umeå University, SE-901 85 Umeå, Sweden; 2 Department of Integrative Medical Biology, Umeå University, SE-901 87 Umeå, Sweden; 3 Umecrine Cognition AB, SE-171 65 Solna, Sweden; 4 Department of Nursing Sciences, Mid Sweden University, AE-851 70 Sundsvall, Sweden

**Keywords:** allopregnanolone, THDOC, GABAA receptor, memory impairment, GR3027 improves memory

## Abstract

Objectives In Vitro: To study the effects of GR3027 (golexanolone) on neurosteroid-induced GABA-mediated current responses under physiological GABAergic conditions with recombinant human α5β3γ2L and α1β2γ2L GABA_A_ receptors expressed in human embryonic kidney cells, using the response patch clamp technique combined with the Dynaflow™ application system. With α5β3γ2L receptors, 0.01–3 μM GR3027, in a concentration-dependent manner, reduced the current response induced by 200 nM THDOC + 0.3 µM GABA, as well as the THDOC-induced direct gated effect. GR3027 (1 μM) alone had no effect on the GABA-mediated current response or current in the absence of GABA. With α1β2γ2L receptors, GR3027 alone had no effect on the GABA-mediated current response or did not affect the receptor by itself. Meanwhile, 1–3 µM GR3027 reduced the current response induced by 200 nM THDOC + 30 µM GABA and 3 µM GR3027 that induced by 200 nM THDOC when GABA was not present. Objectives In Vivo: GR3027 reduces allopregnanolone (AP)-induced decreased learning and anesthesia in male Wistar rats. Rats treated i.v. with AP (2.2 mg/kg) or vehicle were given GR3027 in ratios of 1:0.5 to 1:5 dissolved in 10% 2-hydroxypropyl-beta-cyclodextrin. A dose ratio of AP:GR3027 of at least 1:2.5 antagonized the AP-induced decreased learning in the Morris Water Mase (MWM) and 1:7.5 antagonized the loss of righting reflex (LoR). GR3027 treatment did not change other functions in the rat compared to the vehicle group. Conclusions: GR3027 functions in vitro as an inhibitor of GABA_A_ receptors holding α5β3γ2L and α1β2γ2L, in vivo, in the rat, as a dose-dependent inhibitor toward AP’s negative effects on LoR and learning in the MWM.

## 1. Introduction

The progesterone metabolite allopregnanolone (AP; 3α-OH-5α-pregnan-20-one) and the corticosterone metabolite tetra-hydro-deoxy-corticosterone (THDOC; 3α,21-dihydroxy-5α-pregnan-20-one) are two of the most potent neurosteroids. They act as positive allosteric modulators (PAMs) on the γ-amino-butyric acid (GABA) type A receptor, leading to enhanced GABAergic activity [1,2,3,4], and are therefore called positive GABAA receptor modulating steroids. Both AP and THDOC are sedatives and have similar effects as benzodiazepines, barbiturates, and alcohol [5], and they can be used as anesthetics and cause the loss of the righting reflex [6]. However, the endogenous metabolites bind to the GABA_A_ receptor at specific neurosteroid binding sites, which differs from that of the exogenous GABA_A_ receptor active compounds mentioned above [7,8]. AP and THDOC modulate the post-synaptic GABA_A_ receptor activity in several different aspects [7,8,9]: (1) by prolonging the decay time of spontaneous inhibitory post-synaptic currents (sIPSCs) [10,11] and (2) by causing a direct receptor opening effect (a shift in baseline holding current), especially on extra-synaptic receptors [12,13]. AP also increases the frequency of presynaptic GABA release [10]. In all these aspects, the outcome of AP and THDOC modulation is an augmentation of the chloride ion flux. In general, this leads to hyperpolarization and hence decreased neuronal activity. 

The GABA_A_ receptor is a pentameric ion channel with a multitude of subunit compositions, which have previously been classified [14,15]. The specific subunit composition of each receptor subtype influences the neurosteroid interaction by different degrees of affinity and different effects [16]. It is well known that the α5-GABA_A_ receptor subtype is densely located in the hippocampus and influences learning and memory, while α1 has not been indicated to be as important as α5 for learning and memory. Examples are the mouse mutant lacking the α5 subunit showing enhanced learning [17], and the pharmacological blockade of the GABA_A_ receptor subunit α5 increasing learning and memory [18]. In addition, alpha5IA, a specific α5 inverse agonist on the GABA_A_ receptor, has been shown to improve memory and learning in rodents [19].

It has previously been shown that AP can induce memory and learning deficits in rodents using a test for spatial memory, i.e., in the Morris water maze (MWM) [20,21], and in humans using an episodic memory test [22]. It has also been shown that cognitive function is negatively affected in human conditions when AP levels are elevated, such as in hepatic encephalopathy (HE) [23,24,25], primary biliary cholangitis (PBC) [26], and stress [27,28]. Furthermore, it seems that memory disturbances and dementia can be aggravated or caused by exposure to positive modulators of GABA_A_ receptor active compounds like AP [29], benzodiazepines [30], and alcohol [31]. 

The objective of the present study was to discover a compound that could be used to modulate the steroid-PAM’s negative effect on memory and learning, preferably a compound that could be administered orally. As the α5-GABA_A_ receptor subtype is related to hippocampus and memory performance, the α5-GABA_A_ receptor screening of engineered compounds was performed, subsequently leading to the selection of 3α-ethynyl-3β-hydroxyl-5α-androstan-17-oxime (GR3027, golexanolone) for further characterization and to the results presented in this paper. The chemical structure of GR3027 can be found at Wikipedia (https://en.wikipedia.org/wiki/Golexanolone (accessed on 1 October 2023)). In addition, the structure is given in patent WO 2008/063128.

In these studies, both AP and THDOC were used as PAMs: THDOC in the in vitro experiments and AP in the in vivo studies. THDOC and AP bind to the same binding site on the GABA_A_ receptor and have very similar characteristics. THDOC has a faster washout rate and is therefore more suitable to use in vitro [4,8]. AP is more recognized as a PAM and the dosages suitable for use in studies of cognition are known [20].

It is possible that the AP-induced disturbances of cognitive function can be alleviated if the modulation of GABA activity is inhibited. GR3027 (golexanolone) is a compound related to the endogenous epiandrosterone (3β-hydroxy-5α-androstan-17-one). In the in vitro study, our aim was to investigate whether GR3027 could modulate the THDOC-mediated potentiation of the GABA response on the extra-synaptic α5 GABA_A_ receptor subtype in vitro and investigate the effect of GR3027 on the synaptic α1 GABA_A_ receptor subtype. In vivo, we aimed to investigate whether GR3027 could modulate AP-induced memory disturbances. GR3027 has, to our knowledge, not previously been studied for this purpose and under these circumstances. 

## 2. Materials and Methods

### 2.1. In Vitro Experiments

#### 2.1.1. Transfection of HEK-293 Cells for Electrophysiological Measurements

Wild-type HEK-293 cells (passage 4, originating from Pasteur Institute, Paris, France) were permanently transfected with vectors including the human CMV promoter for the constitutive expression of the human α5, β3, and γ2L GABA_A_ receptor subunits (α5β3γ2L) or the human α1, β2, and γ2L GABA_A_ receptor subunits (α1β2γ2L). The production of the plasmids used in the sub-cloning of the different subunits is published in Rahman et al. [32].

Briefly, total hippocampal RNA isolated from a 78-year-old Caucasian female who had died from congestive heart failure (Ambion) was used for the production of cDNA with Superscript 2 or 3 (Invitrogen^TM^), except for β2, where a whole brain cDNA panel pooled from four male Caucasians with the ages 43–55 years was used (Clontech). The coding regions of the human GABA_A_ receptor subunits were then amplified by PCR with Phusion DNA Polymerase (Finzyme, Espoo, Finland). The primers used were α1 F-AGGCCGCCATGAGGAAAAGTCCAGGT and R-CCCAGTGCAGAGGAACTGAACAACAGA; α5 F-TCTTAGCCGCCATGGACAATG and R-TTGGGAGTGGTGGCCGGTTA; β2 F-ACTGCCGCCATGTGGAGAGTGCGGAA and R-AGGAATCTAGTCCTTGCTTCCAGTGGG; β3 F-AGGAACGCCGCCATGTGCTCCG and R-CAGTCACTCAGTTAACATAGTACAGCCA; and for γ2L F-TGAGCCGCCATGAGTTCACCAAATATA and R-GACTTAGAACAGACATCTCTCCATGAG. A Kozak sequence was introduced shortly before the *ATG* start codon. For cloning, the plasmid pCR^®^-Blunt II-TOPO^®^ and the Zero Blunt^®^ PCR cloning kit were used (Invitrogen^TM^). This was followed by subcloning into mammalian expression vectors: for α1, β5, and γ2L, using the EcoRI restriction sites in pcDNA3.1 (+) for the α subunits and pcDNA3.1/Zeo(+) for γ2L; for β2, using the NotI and BamHI sites; and for β3, the ApaI and KpnI sites in pcDNA3.1Hygro (−) (all vectors from Invitrogen^TM^). Sequences were verified using the Big Dye sequencing kit (Applied Biosystems).

For transfection, wild-type HEK-293 cells cultured in Dulbecco’s Modified Eagle Medium (D-MEM) with GlutaMAX (Gibco), and plasmids purified with the HiSpeed Plasmid Midi Kit (Qiagen) and linearized with PvuI (α1 and α5), BglII (β3), and SspI (β2 and γ2L), were used. Transfections were performed with Lipofectamine^TM^ (Invitrogen^TM^) and Plus^TM^ Reagent (Invitrogen^TM^), one plasmid at a time, starting with that encoding α1 and α5, respectively. A G418-resistant clone was then isolated before transfection with the β2- or β3-containing plasmid, respectively, and cells were grown in the presence of G418 and hygromycin. Cells expressing the GABA_A_ receptor subunit β2 or β3, respectively, on the cell surface were isolated using MACS^®^ cell separation technology (Miltenyi Biotec GmbH, Bergisch, Germany) and anti-GABA_A_ receptor β2/3 monoclonal mouse IgG (Millipore), followed by the isolation of one α1β2 and one α5β3 G418 and hygromycin-resistant clone. These clones were transfected with the γ2L expression plasmid and grown in the presence of G418, hygromycin, and zeocin. MACS^®^ cell separation (Miltenyi Biotec) with rabbit anti-GABA_A_ receptor γ2 antibody (Sigma-Aldrich, St. Louis, MO, USA) was followed by the isolation of α1β2γ2L and α5β3γ2L G418-, hygromycin- and zeocin-resistant clones. The cell lines produced were analyzed for the three GABA_A_ receptor subunits using immunocytochemistry, followed by the selection of a suitable cell line showing good reactivity to GABA and THDOC. The transfected cells were used for patch clamp experiments at the earliest after two passages of thawing and 3–5 days after seeding. The cells were seeded 20–24 times for different receptor subtypes.

#### 2.1.2. Electrophysiological Recordings

The electrophysiological recordings from HEK-293 cells were performed with the whole-cell voltage clamp technique, at room temperature (21–23 °C). After compensation for liquid junction potential, a steady holding potential of −17 mV was used in all experiments. Patch pipettes were pulled from borosilicate glass (Bergman Labora AB, Danderyd, Sweden) and polished to a resistance of 2–5 MΩ when filled with intracellular solution and immersed in a bath (extracellular) solution (for solutions, see below). The pipette tips were filled by immersion in standard intracellular solution and subsequent back-filling with intracellular solution. The recordings were made using an Axopatch 200B amplifier and a Digidata 1322A (Axon Instruments, Foster City, CA, USA) controlled via a Pentium processor-based personal computer. Data were acquired using the pCLAMP software sampled at 10 kHz, filtered at 2–10 kHz, and analyzed with Clampfit (versions 9.0, both from Axon Instruments, Foster City, CA, USA). A series resistance between the pipette and cell membrane not higher than 20 MΩ was accepted. No series resistance compensation was used. The stability of series resistance was monitored repeatedly from the time course of capacitative transients during the experiments. The measured liquid junction potential between extracellular and intracellular solutions was subtracted from all data presented, as previously described [33]. All experiments were performed at room temperature (21–23 °C). 

HEK-293 cells placed in a bath filled with extracellular (control) solutions were visually guided and patched using an inverted Zeiss Axiovert 25 microscope equipped with an objective (×20). In all experiments, the Dynaflow™ system (see below) was exploited for the quick and precise application of control and test solutions to patched cells. 

#### 2.1.3. Experimental Solutions, GABA and Neurosteroids

The standard extracellular (bath/control) solution contained (in mM) NaCl 137, KCl 5.0, CaCl_2_ 1.0, MgCl_2_ 1.2, HEPES 10, and glucose 10. The pH was adjusted to 7.4 with NaOH, ~310 mOsm. The standard intracellular solutions, used for the filling of pipettes contained (in mM) Cs-gluconate 140, NaCl 3.0, MgCl_2_ 1.2, EGTA 1.0, HEPES 10, Mg-ATP 2 (pH was adjusted to 7.2 with CsOH, ~305 mOsm) or Cs-gluconate 140, NaCl 3.0, MgCl_2_ 1.2, EGTA 1.0, and HEPES 10 (pH was adjusted to 7.2 with CsOH, ~301 mOsm). A series of experiments were performed using IC without ATP. Although the deterioration of cell conditions was comparably faster in cells patched without ATP, neither the response to GABA stimulation nor the effect of neurosteroids differed significantly from the effects observed in experiments with ATP-containing IC.

In all electrophysiological experiments, tetrahydro-deoxycorticosterone (THDOC) was used as a positive GABA_A_ receptor steroid modulator (PAM) to mimic the effect of AP on central GABA_A_ channels [4]. THDOC and AP bind to the same binding site on the GABA_A_ receptor but THDOC has a faster washout rate and is more suitable for use in vitro [4,8]. THDOC (Sigma Chemical Co., St. Louis, MO, USA) and GR3027 (3α-ethynyl, 3β-hydroxyl, 5α-androstan-17-oxime, golexanolone, Umecrine Cognition AB, Stockholm, Sweden) were dissolved in dimethyl sulfoxide (DMSO) using ultrasound, for the preparation of solutions containing a final DMSO concentration of 0.1%. GR3027 was discovered within a series of molecules developed and patented by T. Bäckström and G. Ragagnin, (patent WO 2008/063128). All solutions were complemented with DMSO to achieve the same final concentration as in the steroid-containing test solution. GABA (Sigma Chemical Co., St. Louis, MO, USA) was first prepared as a stock solution (dissolved in EC with ultrasonic treatment) to the desired concentrations, with the corresponding final concentrations of test solutions (30 µM and 0.3 µM; see protocols below). GABA was added to the experimental solution without a change in osmolarity. 

#### 2.1.4. Dynaflow™ System

The Dynaflow™ system (Dynaflow Pro II Platform Zeiss Axiovert 25; Fluicell AB, Cellectricon AB, Sweden) with Resolve chips was used for all patch clamp experiments. The Resolve chip consists of a glass chip enclosed by a PEEK plastic top part and a supporting plastic bottom part. Both the wells and the channels are glass-coated. The channel width is 150 µm and the height is 50 µm. The well volume is 150 µL. The run time at the flow rate of 26 µL/min is 90 min. The pump settings were as follows: a BD Plastikpak^TM^ 2 mL syringe with inner diameter of 8.1 mm was used. The syringe pump flow rate for the Resolve chip was 26 µL/min. The Resolve chip allows the synchronized control of switching between 16 experimental solutions. Laminar flow at each solution outlet of the microfluidic chip prevents mixing, and a computer-controlled stage motor is used to move the chip relative to the patch pipette, allowing relatively rapid solution exchange around the membrane patch. 

#### 2.1.5. Electrophysiological Protocol

The experimental protocols were designed to mimic the physiological conditions of the tonic (extra-synaptic) and phasic (synaptic) activation of GABA_A_ receptors observed in central neurons, depending on the subunit compositions, respectively. GABA-evoked currents in *HEK-293* cells expressing extra-synaptic (α5β3γ2L) or synaptic (α1β2γ2L) types of GABA_A_ receptors were studied using the conventional whole-cell patch clamp technique. To mimic the physiological GABA exposure, the *HEK-293* cells expressing α1β2γ2L GABA_A_ receptors were exposed to short applications (40 ms) of 30 µM GABA, whereas *HEK-293* cells expressing α5β3γ2L GABA_A_ receptors were exposed to prolonged applications (6 s) of 0.3 µM GABA, either in the absence or presence of THDOC and/or GR3027. To evaluate the effect of neurosteroids on the GABA_A_ receptor-mediated response, *HEK-293* cells were challenged with THDOC alone or in combination with GR3027 for 20 s prior to and during GABA application. The application of all tested solutions was followed by at least 2 min of washout with a control solution.

The reason behind this approach was the goal of gaining insights solely into the actions of GR3027 on the potential physiological effects of THDOC and to prepare for in vivo investigations. The aim was thus to investigate whether GR3027 functions as an inhibitor/antagonist under physiological conditions of GABA and THDOC stimulation on the *α5β3γ2L* type of receptor and α1β2γ2L type of GABA_A_ receptor. The aim was not to investigate whether GR3027 had any subtype-specific characteristics, such as being more active on the *α5β3γ2L* GABA_A_ receptor than on the *α1β2γ2L* GABA_A_ receptor. The aim was to determine whether GR3027 showed GABA_A_ modulating steroid antagonism on both a memory-linked extra-synaptic receptor type (*α5β3γ2L)* and a more general intra-synaptic receptor type (*α1β2γ2L*) [34].

However, to further elucidate the interaction between THDOC and GR3027 in the extra-synaptic (α5β3γ2L) type of GABA_A_ receptor, the effect of 10–1000 nM THDOC in the presence of 0.3 µM GABA +/− 1 and 3 µM GR3027 was measured as the AUC, % change in 0.3 µM GABA. To evaluate the interaction between THDOC and GR3027 antagonism in the absence of GABA, the mean current response, pA, compared to the current response at EC buffer application (baseline holding current) was measured. 

#### 2.1.6. Data Analysis

Data analysis and graphical presentation were performed using the Clampfit^TM^ data analysis module (Molecular Devices, LLC, Sunnyvale, CA, USA), the SPSS statistical package (IBM Corp., Armonk, NY, USA), Microsoft Excel (Microsoft Corp., Redmond, WA, USA), and the Origin package (OriginLab Corp., Northampton, MA, USA). Dose–response curves and EC_50_, IC_50_, and IC_max_ values were analyzed using the GraphPad Prism software (GraphPad Software Inc., La Jolla, CA, USA). The logistic equations used were (1) I = I_max_/(1 + (EC_50_/C)^n^), where I is the current, with subscript “max” denoting the maximum current, C denoting the concentration of THDOC, EC_50_ the half-saturating concentration, and n = the “Hill slope”; and (2) I = I_min_ + (1 − I_min_)(1 + (C/IC_50_)^n^), where I is the current, with subscript “min” denoting the minimum current, C denoting the concentration of GR3027, IC_50_ the half-blocking concentration, and n the “Hill slope”. Evoked currents elicited by the external application of the test solution (GABA and/or THDOC alone or in combination with GR3027, as specified in the electrophysiological protocol below) were analyzed off-line using Clampfit. The peak current and area under curve within a 20 ms (*α1β2γ2L*) or 10 s (*α5β3γ2L)* time window (starting at the application of test solution onset) were detected for each response. The mean baseline holding current during the 5 s interval preceding the inflection point at the start of the evoked current was considered as a reference point for the measured parameters. As a rule, currents with an amplitude less than 10 pA were discarded from analyses. The data are presented as mean ± SEM, unless stated otherwise. The Kruskal–Wallis test and Wilcoxon matched-pairs signed-rank test followed by Bonferroni adjustment were used when appropriate for statistical evaluation, with effects considered significant when *p* ≤ 0.05 (*), *p* ≤ 0.01 (**), and *p* ≤ 0.001 (***).

The paired non-parametric Wilcoxon signed-rank test followed by Bonferroni adjustment was used to evaluate the significance of the THDOC effect ≥10 pA, compared to the baseline holding current with the EC solution. At concentrations of THDOC that significantly increased the current response, the reducing effect of GR3027 was calculated for significance. The paired non-parametric Wilcoxon signed-rank test followed by Bonferroni adjustment was used for THDOC vs. THDOC + GR3027. 

### 2.2. Animal Experiments

#### 2.2.1. Studies of AP on Dissociated Singe Cells from Hypothalamus

For the patch clamp studies, male Sprague-Dawley rats (n = 5, weight < 130 g) were housed at two or four rats per cage at 22 °C and with artificial daylight. Rats had free access to water and standard food. The regional ethics committee for animal research approved the experimental protocols. All procedures including animals were conducted in accordance with the ethical guidelines set by the European Union Directive 2010/63/EU for animal experiments. 

*Drugs and chemicals:* Allopregnanolone (AP; 3α-OH-5α-pregnane-20-one) was obtained from Umecrine AB (Umeå, Sweden). GABA and amphotericin B were obtained from Sigma Chemical Co. (St. Louis, MO, USA). Other chemicals used were purchased from a local supplier.

*Electrophysiological experiments:* The methods used for electrophysiological recording and cell preparation have been described in detail elsewhere [10,11,12,13]. A summary of the procedures is given below. The oxygenated extra cellular solution (EC) contained a high Cl^−^ concentration, while the intracellular solution (IC) in the patch clamp pipette contained a low Cl^−^ concentration; for details, see Das et al. [13]. In this study, the intracellular chloride concentration in the patch clamp pipette was [2.4 mM Cl^−^], while the extracellular chloride concentration was [125.0 mM Cl^−^]. AP was dissolved by sonification in the EC solution, with DMSO as a solvent. The dosages of AP used in the patch clamp experiments were 0–100 nM. 

*Cell preparation:* The rats were decapitated, without using anesthesia. A block of tissue containing the hypothalamus was dissected. Brain tissue slices (thickness of 250 µm) were prepared and incubated for 1 h in oxygenated incubation solutions at 28 °C. Single cells with adhering synaptic nerve terminals from the medial preoptic area of the hypothalamus were isolated by vibro-dissociation [10]. The cell bodies of dissociated neurons were round/oval, and the preparation contained only the cell body with presynaptic terminals; no axons remained in the preparation [10,11,12,13].

*Electrophysiological recordings:* The amphotericin-B-perforated patch technique [11] was used to record whole-cell currents from the postsynaptic neurons under voltage clamp conditions at room temperature (21–23 °C). Cells were bathed in an EC solution and the patch pipettes were filled with IC. The steady voltage used for recording from preoptic neurons was (after correction for liquid junction potential) –11 mV. The equilibrium potential for Cl^−^ in these recordings was –100 mV. EC-solutions, with or without test substances, were applied by a fast perfusion system controlled by solenoid valves. Borosilicate glass pipettes (GC150, Clark Electromedical Instruments, Pangbourne, UK), with a resistance of 3–4 MΩ, were used.

*Protocol and analysis:* A standard protocol was used for the experiments. Firstly, the current was recorded in the control solution (EC solution) for 30–60 s, followed by a 60–300 s test period with or without AP. The spontaneous miniature inhibitory postsynaptic currents (mIPSCs) were detected by visual inspection of current changes. The test period was followed by a 2–4 min washout period in the control solution. The protocol was applied repeatedly for recordings from individual cells. The time-course and amplitude of the currents were measured semi-manually by using cursors and were fitted to an exponential decay curve by the curve-fitting routine of the pCLAMP software (Axon Instruments, Foster City, CA, USA). The peak amplitudes and area under the curve (AUC) of the currents were analyzed for events of amplitude ~1.5 times the peak-to-peak noise (in the solution with the largest noise). All the mIPSC values from the treatment period of the recorded cell (n = 6–11 events) were pooled and the mean was subjected to statistical analysis. 

*Statistical analysis:* The data analysis of concentration–response data was non-parametric to compare the AP effect with the control value. The SPSS statistical package version 26.0 (SPSS Inc., Chicago, IL, USA) was used for all tests.

#### 2.2.2. Studies of AP’s Effect on Loss of Righting Reflex

Nanomolar concentrations of AP injected intravenously dramatically potentiate the GABA response at the GABA_A_ receptor [1,20]. In this respect, AP is one of the most effective anesthetics known [6] and can thus be used to induce the loss of the righting reflex (LoR), i.e., to cause unconsciousness. Under normal circumstances, the righting reflex immediately corrects the orientation of the body when it is outside of its normal upright position, such as that of a rat that is placed on its side. When the rat is awake, it immediately turns around to be standing on its paws when dropped from a low distance above a surface. This ability can be lost with the injection of AP. Twelve [12] male Wistar rats, with a weight of 267–307 g at the test occasion, were included in the study (Taconics, Denmark). The rats were housed at three animals per cage, with a normal day/night light cycle and ad libitum access to food and water (except during the short experiment, approximately 10 min). The week before the study, the animals were handled daily, to avoid stress during the experimental session. The handling included activities used during the test, i.e., holding, tail warming in water, and wrapping in op-blankets. AP (Umecrine AB) and GR3027 (Umecrine Cognition AB) were dissolved at a concentration of 2 mg/mL in 10% 2-hydroxypropyl-β-cyclodextrin (Sigma), respectively, by sonication. To measure the antagonism of AP-induced LoR, GR3027 was mixed with the AP solution at different ratios of AP to GR3027 1: 2.5–1:7.5. All procedures including animals were conducted in accordance with the ethical guidelines set by the European Union Directive 2010/63/EU for animal experiments, and the study protocol was approved by the Regional Ethics Committee of Umeå University, Sweden. 

#### 2.2.3. Studies of AP Effect on Learning and Spatial Memory

*Animals:* Male Wistar rats were used to study the effect of AP on learning and spatial memory (Scanbur BK, Sweden). The rats were marked on the tail with a permanent spray (Porcimark™) for identification. A 12/12 h light/dark cycle was used (lights off at 18.00 h), food and water were available ad libitum, and the housing room temperature was kept at 22–23 °C. Animal testing was performed during the light part of the day. After arrival at the animal facility, the handling of all rats was performed for two weeks, including habituation to all relevant experimental procedures. Eighty-three rats (83 + 6 satellite rats for vehicle treatment) were used for the MWM experiment (weight at arrival was 207–250 g, and that at testing was 250–340 g), which were housed in pairs. In total, 24 rats were used for the pharmacokinetic study (weight at arrival: approximately 150 g; at testing: 250–320 g), which were housed four in each cage. The animal experiments were performed according to EU Directive 2010/63/EU and the study protocol was approved by the Regional Ethics Committee of Umeå University, Sweden.

*Spatial memory:* Spatial memory was assessed using the Morris water maze (MWM), as previously described in detail [20], and in brief as follows. The pool Ø was 180 cm, the platform Ø was 10 cm, and the water temperature was 25 °C. The animals were habituated twice to the swim situation, each time by a one-minute swimming session without the platform present in the pool. All animals performed swim tests of four swims per day for five consecutive days. Each swim lasted until the platform was found or maximally 120 s and started at different positions in a randomized order. If unsuccessful, the rats were shown to the platform. The rats remained for 30 s on top of the platform before being rescued and dried with a towel during a 30 s interval before the next swim. After the final swim, the rats were returned to their home cages. MWM parameters (latency, path, swimming speed, floating, and thigmotaxis) were monitored and quantified using the computer tracking system Menu 2020 (HVS Image, UK). Mean values for each day of testing were used for statistical comparisons.

*Substances and treatments:* AP (3α-OH-5α-pregnan-20-one; 2.0 mg/mL; Umecrine AB, Sweden) and GR3027 (3α-ethynyl-3β-hydroxyl-5α-androstan-17-oxime; 2.0 mg/mL; Umecrine Cognition AB, Stockholm, Sweden) were dissolved in 10% 2-hydroxypropyl-beta-cyclodextrin (β-CD; Sigma, St. Louis, MO, USA) using ultrasound. The various combination treatments were then prepared by mixing these solutions in calculated proportions (Table 1). 

The animals were randomized into six treatment groups (Table 1). All treatment groups were administered i.v. injections (4 mg AP/min or equivalent volume/min when the solution did not include AP), which were completed 8 min before the start of the MWM testing, during which time each rat was in a separate cage. For comparisons, one group of rats was treated with only the vehicle, 10% 2-hydroxypropyl-beta-cyclodextrin (β-CD; Sigma, St. Louis, MO, USA). 

*Statistical analysis of MWM data:* Statistical data analyses were performed using the statistical software SPSS Statistics (IBM Corp., Armonk, NY, USA), and the area under the curve was calculated using the GraphPad Prism software (GraphPad Software Inc., La Jolla, CA, USA). *p* values of <0.05 were considered statistically significant. All values in the text are displayed as mean ± SEM unless otherwise stated.

A 2-way ANOVA repeated-measures analysis was used to determine the effects on the MWM learning and behaviors. This included the test of within-subjects effects over time and the test of within-subjects effects across the interaction between time and treatment: the Sphericity Assumed test when Mauchly’s test of sphericity was not significant and the Greenhouse–Geisser test when Mauchly’s test of sphericity was significant, respectively. Differences in between-subjects and within-subjects effects and the treatment groups were determined using ANOVA, followed by the least significant difference test as a post hoc test. 

#### 2.2.4. Pharmacokinetic Analysis of GR3027

Ten male Sprague-Dawley rats were used to assess PK with GR3027 given orally and intravenously, respectively. Animals were kept at room temperature with artificial light under a 12/12 h light/dark schedule, and with food and water ad libitum. Animals were given 2 mg/kg i.v. in 10% 2-hydroxypropyl-beta-cyclodextrin or 10 mg/kg orally. Approximately 50 µL blood samples were taken at 15 min, 30 min, 1 h, 2 h, 4 h, 8 h, 12 h, and 24 h after oral dosing, and 5 min, 15 min, 30 min, 1 h, 2 h, 4 h, 8 h, 12 h, and 24 h after intravenous dosing, and plasma was frozen. The analysis method has been described earlier and is here briefly described [35]. All the samples were analyzed in triplicate by Admescope Ltd., Oulu, Finland, using an LC/MS/MS method developed for the purpose, using a Waters ACQUITY UPLC_Waters XEVO-TQS triple-quadrupole mass spectrometer.

## 3. Results

### 3.1. In Vitro Studies

#### 3.1.1. THDOC Effects on Extra-Synaptic α5β3γ2 GABA_A_ Receptors

**Figure 1:**
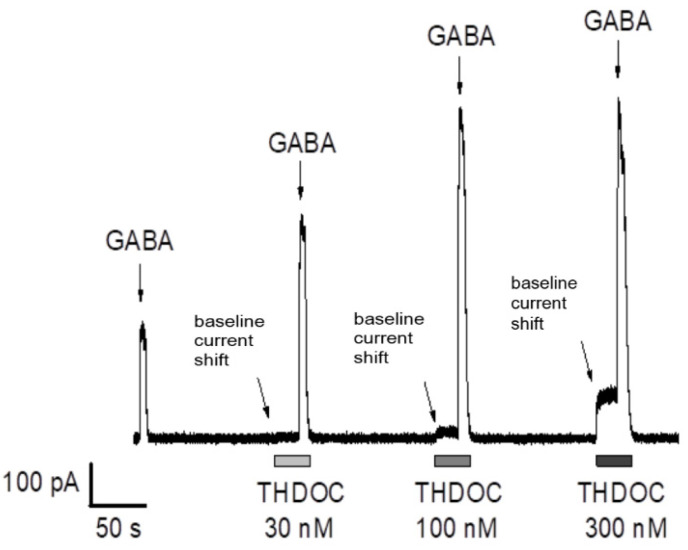
Dose-dependent effect of THDOC on GABA-mediated currents in α5β3γ2L GABA_A_ receptors. THDOC increased the amplitude and prolonged the decay of the responses to 0.3 µM GABA (relative to the first peak without THDOC application). Note the outward shift in baseline holding current when THDOC (30–300 nM) is applied (see arrows pointing to the shift). α5β3γ2L-expressing HEK-293 cells were exposed to a series of prolonged applications of low-concentration GABA (6 s, 0.3 µM GABA) to mimic the tonic activation of extra-synaptic GABA_A_ receptors. Except for the first application, all GABA applications in each series were performed in combination with THDOC, delivered in incremental concentrations ranging from 30 to 300 nM. Each application of GABA and THDOC in combination was preceded by a 20 s exposure to THDOC only. The current responses were continuously recorded with the holding membrane potential −17 mV (corrected for the liquid junction potential).

To mimic the physiological tonic activation of human extra-synaptic GABA_A_ receptors, α5β3γ2L-expressing HEK-293 cells were exposed to a series of prolonged applications of low-concentration GABA (6 s, 0.3 µM GABA). Except for the first application (GABA alone), all GABA applications in each series were performed in combination with THDOC delivered in incremental concentrations ranging from 30 to 300 nM. The application of THDOC induced an outward shift in the baseline holding current, suggesting the direct activation of α5β3γ2L GABA_A_ receptors (Figure 1, see arrows indicating the shift in baseline holding current when THDOC is applied). The effect was concentration-dependent, with EC_50_ = 32 nM and E_max_ = 145% (in agreement with previously published data on the sensitivity of GABA_A_ receptors to THDOC; [36]). As previously published by us and others, THDOC dramatically potentiated the α5β3γ2L GABA_A_ receptor-mediated currents when applied with GABA [36]. Analysis of the data revealed a significant increase upon THDOC application in both the peak current amplitude and the area under the current curve. Thus, the results demonstrate that, in our setting, THDOC may directly activate α5β3γ2L GABA_A_ receptors expressed in HEK-293 cells and positively modulate their responses to GABA application. To be able to identify GR3027 and THDOC’s effects both on the amplitude and the area under the amplitude curve and the shift in baseline holding current, a dosage of 200 nM THDOC was optimal but still relevant to physiological concentrations [37].

#### 3.1.2. GR3027 Effects on THDOC-Mediated Potentiation of Extra-Synaptic α5β3γ2L GABA_A_ Receptors

To examine whether GR3027 may affect the THDOC-mediated potentiation of α5β3γ2L GABA_A_ receptor activation, current responses to THDOC with or without GABA in physiological concentrations were recorded under the same protocol conditions as described above with the concentration of THDOC set to 200 nM. The relatively high THDOC concentration (200 nM) as compared to the obtained EC_50_ = 32 nM was chosen based on in vivo studies showing that the level of THDOC in the brain significantly varies depending on physiological conditions such as stress, menstrual cycle, and pregnancy [37,38]. 

**Figure 2:**
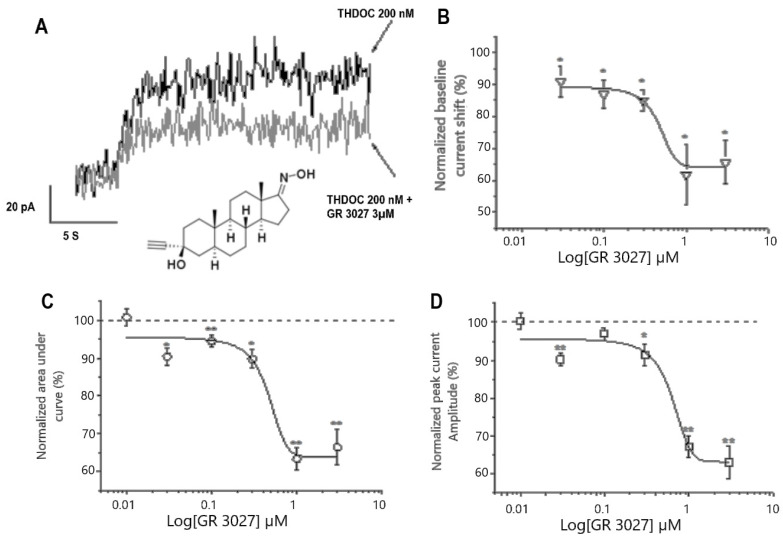
GR3027 attenuates THDOC-mediated potentiation of α5β3γ2L GABA_A_ receptors. (**A**). Baseline holding currents recorded from HEK-293 cells, clamped at −17 mV. Note the THDOC-mediated outward current shift in the absence (black) and in the presence (gray) of GR3027 (3 µM), as indicated. Inserted in (**A**) is the structure of GR3027. (**B**). Concentration–response relation for GR3027-induced depression of THDOC-mediated outward current shift. Mean currents measured after THDOC (200 nM) application in the presence of GR3027 (0.01–3 µM). Smooth line is described by best sigmoidal fitting. SEM ± SE, data from 4–14 cells. (**C**,**D**). Concentration–response relation for GR3027-induced depression of THDOC-mediated GABA responses for AUC (**C**) and peak current amplitude (**D**). Mean currents measured after THDOC (200 nM) application in the presence of GR3027 (0.01–3 µM). Smooth line is described by best sigmoidal fitting. SEM ± SE. Data from 4–14 cells. * Different from 100%, *p* < 0.05; ** different from 100%, *p* < 0.01.

In the absence of an agonist (i.e., GABA, 0.), the outward THDOC-mediated current shift was significantly reduced when GR3027 was applied in incremental concentrations (0.03–3 µM, Figure 2A). The effect was dose-dependent (Figure 2B), with IC_50_ = 344 nM and I_max_ −33%. Moreover, the analysis of the AUC and the peak current amplitude of the GABA-mediated responses revealed a concentration-dependent decrease in both parameters (IC_50_ = 341 nM and I_max_ −35% for AUC and IC_50_ = 563 nM and I_max_ −37% for peak current amplitude; n = 4–14, 95% confidence interval; Figure 2C,D). Thus, our data demonstrate that GR3027 had a prominent inhibiting effect on the THDOC-mediated activation of the α5β3γ2L GABA_A_ channels.

#### 3.1.3. Studies on the Interaction between THDOC and GR3027 in α5β3γ2L-GABA_A_ Receptors 

**Figure 3:**
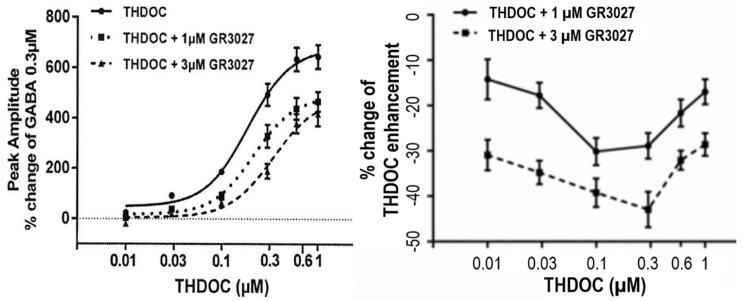
(**Left**) With α5β3γ2L GABA_A_ receptors, the effect on peak amplitude of THDOC in concentration interval 0.01 to 1 µM and 0.3 µM GABA, investigated +/− presence of 1 µM or 3 µM GR3027. GABA 0.3 µM was present in all tests as the GABA_A_ receptor activator. Moreover, 0.3 µM GABA was the control, set as 0%. (**Right**) The effect of GR3027 as % change in THDOC enhancement related to increasing THDOC concentration shows that 3 µM GR3027 antagonizes THDOC enhancement more than 1 µM GR3027 (*p* = 0.000, **right**).

To determine the effect of different dosages of GR3027 on the THDOC-enhanced GABA-mediated current response mediated by 0.3 µM GABA and THDOC in a concentration interval of 0.01–1 µM, the dosages of GR3027 analyzed were 1 µM and 3 µM. It was found that THDOC in the concentration interval of 0.01–1 µM increased the 0.3 µM GABA-evoked current response in a concentration-dependent manner (*p* = 0.000). The concentration-dependent increase occurred also with THDOC + 1 µM GR3027 (*p* = 0.000) and THDOC + 3 µM GR3027 (*p* = 0.000) (Figure 3, left). The effect was calculated as the % change in peak amplitude at 0.3 µM GABA. The two different concentrations of GR3027 1 µM and 3 µM were tested in separate experimental runs with their own THDOC control curves—first, THDOC +/− 1µM GR3027, trial 1, and THDOC +/− 3µM GR3027, trial 2. In in the figure, the two THDOC curves are merged (Figure 3). The THDOC concentration response curve shows a THDOC EC_50_ = 0.19 µM and E_max_ = 687%, while THDOC + 1 µM GR3027 EC_50_ = 0.22 µM and E_max_ = 489% and THDOC + 3µM GR3027 EC_50_ = 0.35 µM and E_max_ = 482% (Figure 3). 

To further clarify the antagonism and concentration dependence of GR3027, the effect was also calculated as the % change in THDOC enhancement. At both 1 µM and 3 µM, we found that the concentration-dependent effect of GR3027 was not dependent on the concentration of THDOC. Further, these data show that 3 µM GR3027 antagonized the THDOC enhancement more effectively than 1 µM GR3027 (*p* = 0.000, Figure 3 right).

#### 3.1.4. GR3027 Effects on Extra-Synaptic α5β3γ2L GABA_A_ Receptors without the Presence of THDOC or GABA

Next, to clarify whether the observed inhibiting effect of GR3027 was mediated by a direct inhibitory effect on its own on the GABA_A_ receptors, the baseline holding current and GABA-mediated currents were monitored at the same conditions as described above but in the absence of THDOC. In this series of experiments, GR3027 was used at the concentration of 1 µM. When applied in the absence of THDOC, GR3027 did not change the baseline holding current, suggesting no direct influence on the GABA_A_ receptors at the concentration of 1 µM. Further analysis demonstrated that the peak current amplitude of the 0.3 µM GABA-mediated responses was significantly decreased by 1 µM GR3027 (mean change −7.0%, SEM 0.9, *p* = 0.02, n = 7), while the AUC remained unaffected (mean change −3.8%, SEM 1.5, NS, n = 7), suggesting that the probability of a channel open state was not influenced by GR3027. This suggests that GR3027 alone has no direct gating effect on the GABA_A_ channel function within the tested concentration range.

**Figure 4:**
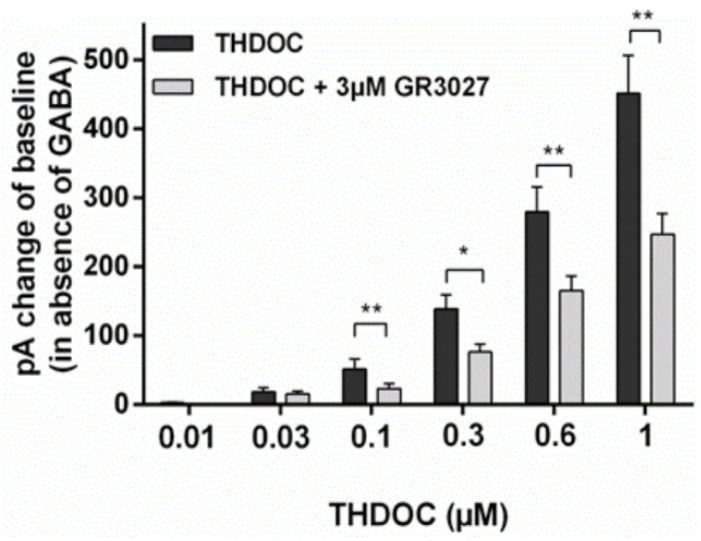
The effect of GR3027 on THDOC-induced current response (in absence of GABA) at α5β3γ2L receptor. pA = pico Ampere, * = *p* < 0.05, ** = *p* < 0.025.

To investigate whether GR3027 could antagonize THDOC’s direct activation of the α5β3γ2L GABA_A_ receptor, we investigated the change in current response (pA) in the presence of THDOC and THDOC + 1 µM GR3027, as well as THDOC and THDOC + 3 µM GR3027 (Figure 4). The THDOC-induced current responses were compared to the control, the EC solution (trial 1: +0.54 pA ± 0.58 (N = 32); trial 2: −0.24 pA ± 0.45 (N = 37)). A change in current response of less than 10 pA was not included in the statistical calculation. At concentrations of THDOC that significantly increased the current response, the reducing effect of GR3027 was calculated for significance. Here, 1 µM GR3027 significantly reduced the 1 µM THDOC-induced current response, while 3 µM significantly reduced the induced current response by 0.1, 0.3, 0.6, and 1 µM THDOC.

The effect of 1 and 3 µM GR3027 alone on the α5β3γ2L GABA_A_ receptor was investigated without GABA or THDOC present in the solutions. GR3027 did not activate the GABA_A_ receptor by itself (baseline holding current +2.15 ± 1.4 pA n = 9, current at 1 µM GR3027 +1.6 ± 1.4 pA, and 3 µM GR3027 +0.73 ± 0.4 pA, n = 9; both 1 and 3 µM dosages were not significantly different from baseline holding current). 

#### 3.1.5. GR3027 and THDOC Effects on Synaptic α1β2γ2L GABA_A_ Receptors

The current responses recorded from HEK-293 cells expressing α1β2γ2L GABA_A_ receptors were investigated to examine whether GR3027 could affect synaptic GABA_A_ receptor function when modulated by THDOC. The experimental protocol was designed to mimic synaptic events under physiological conditions. Therefore, the currents were evoked by the rapid and short application of a saturating concentration of GABA (40 ms, 30 µM using the Dynaflow^®^ equipment) and monitored in the presence of 200 nM THDOC and 0.1–3 µM GR3027. The THDOC-induced GABA responses were attenuated at GR3027 concentrations in the range of 1–3 µM, while, at lower concentrations, no reduction in the GABA currents was observed. The analysis of the AUC at 1 and 3 µM GR3027 application showed a significant reduction (AUC: 3 µM GR3027 + THDOC = −22.9 ± 2.8 and THDOC + 1µM GR3027 = −11.6 ± 3.1, n = 8, *p* ≤ 0.05, respectively). A significant decrease in the THDOC-mediated baseline holding current shift in the absence of GABA was also observed at the highest GR3027 concentration of 3 µM (−38.1 ± 7.6%, n = 7, *p* ≤ 0.05). No difference in baseline holding current or 30 µM GABA-mediated currents was observed during the application of GR3027 alone using a similar protocol as above (GR3027 (1 µM) + GABA (30 µM), AUC, –3.1 ± 1.7% (NS; *n* = 21)). Thus, GR3027 antagonized the THDOC’s potentiation of GABA’s effect on the α1β2γ2L GABA_A_ receptor, without having a direct gating effect on the GABA_A_ channels, in the GABA and THDOC dosages used in the present experimental conditions, reflecting physiological dosages.

### 3.2. In Vivo Animal Studies

#### 3.2.1. AP Effects on Single Hypothalamic Cells Obtained In Vivo

**Figure 5:**
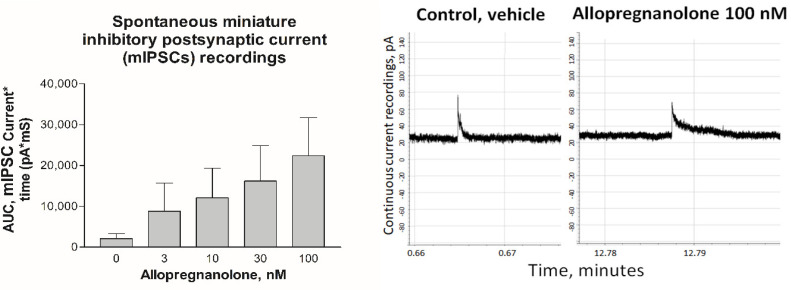
On the left: Mean (SEM) area under the curve (AUC) of spontaneous miniature inhibitory postsynaptic currents measured with whole cell patch clamp (holding potential −11 mV, n = 5) dissociated hypothalamic cells. The recordings were performed during vehicle perfusion and during increasing AP dosages. The responses at 30 and 100 nM were significantly higher than in the vehicle (*p* < 0.05). On the right: Traces of continuous recordings of spontaneous miniature inhibitory postsynaptic current (mIPSC) responses to GABA release from one hypothalamic cell during infusion of vehicle (**left** figure) and recording during infusion of 100 nM AP (**right** figure). The recording started at 0 min, and, between the control period and AP infusion, there were several washout events using the vehicle. Note the prolonged closing duration during the AP infusion.

The in vitro studies were performed using THDOC, while the in vivo studies were performed using allopregnanolone (AP). To bridge the dose–effect relation between THDOC and AP, we therefore tested the effect of low doses of AP in an in vitro system close to in vivo conditions, namely in dissociated hypothalamic cells from male rats [11,13]. We noted that there was a significant effect on the spontaneous miniature inhibitory postsynaptic currents (mIPSCs) at a concentration of 30 nM and 100 nM in the hypothalamic cells (Figure 5). We noted that the dose effects of THDOC and AP were in the same range and that the dosages used in the in vitro studies were in the physiological range. We also noted that the AP dosages had effects on the hypothalamic cells (Figure 1 and Figure 5). In earlier studies, we found, after dose–response analyses, that an AP i.v. dose of 2 mg/kg is suitable to identify memory and learning disturbances but that it does not induce sedation or anesthesia [20]. We noted the concentration received by i.v. injections of AP giving acute memory and learning disturbances to be around 1–2 µM in brain tissue [20]. In humans, the normal physiological brain concentration of AP is up to 60 nM in women [37]. 

#### 3.2.2. GR3027 and AP Effects on Loss of Righting Reflex

The antagonizing effect of GR3027 on the positive GABA_A_ receptor-modulating steroid AP was tested in vivo in male Wistar rats. AP given i.v. at 2 mg/kg induced the loss of the righting reflex (LoR) and GR3027 was evaluated against the LoR induced by AP alone. The results showed that GR3027 at a dosage of 15 mg/kg could antagonize 2 mg/kg of AP. This gives an AP:GR3027 ratio of 1:7.5. Lower concentrations of GR3027 did not, under these circumstances, inhibit APs effect on LoR. GR3027 at doses of 15 mg/kg did not affect the general condition of the rat. 

#### 3.2.3. GR3027 and AP Effects on Spatial Memory

**Figure 6:**
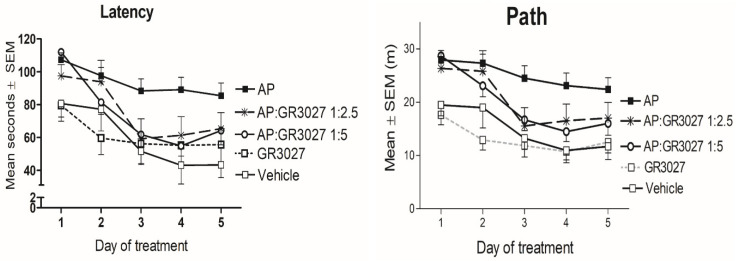
The AP-induced learning impairment was alleviated by GR3027 treatment in the MWM trial. Data are shown as mean ± SEM for latency, time (seconds) until they found the platform/day of testing (**left** picture). On the (**right**) is the swim path in the Morris water maze until the platform was found, for the treatment groups, during the five days of testing. Treatments were vehicle (10% Captisol; n = 6), AP (2.2 mg/kg in β-CD; n = 20), AP:GR3027 1:2.5 (2.2:5.5 mg/kg in β-CD; n = 10), AP:GR3027 1:5 (2.2:11 mg/kg in β-CD; n = 15), and GR3027 (11 mg/kg in β-CD; n = 11). Daily i.v. injections were administered 8 min before each rat’s first swim of the day. For statistically significant differences, see Table 2 for *p*-values.

To investigate whether GR3027 could modulate AP-induced deficits in spatial memory in the MWM, rats were given a combined treatment including both AP and GR3027 with various ratios of the latter compound ranging from 1:0.5 to 1:11 (T1:1 to T1:11). Control groups were given AP only, GR3027 only, or a vehicle, respectively (Figure 6). All treatment groups improved as expected during the MWM learning phase, i.e., all groups had a significantly shorter latency (Table 2A) and path (Table 2B) over time (*p* < 0.05 for individual groups’ repeated-measures analyses). However, the group given AP treatment had a significantly longer latency (Table 2A; Figure 6 left) and path (Table 2B; Figure 6 right) to find the platform compared to the group given vehicle treatment. The latency and path of the group given GR3027 alone did not differ compared to the vehicle group, and the GR3027 group had a significantly shorter latency and path than the AP group. The groups given the treatments combining AP and the two higher doses of GR3027 (T1:5 and T1:2.5), respectively, had a significantly shorter latency than the group given AP only, while the latency and path did not differ compared to the vehicle or GR3027 alone groups, respectively. The T1:5 and T1:2.5 groups had also shorter paths than the vehicle group, but significantly so only for the group given the T1:5 treatment. The groups given the treatments combining AP and the two lower doses of GR3027 (T1:1 and T1:0.5), respectively, did not differ in latency and path compared to the group given AP and had a significantly longer latency and path than the groups given the vehicle and GR3027, respectively. These data suggest that the AP-induced spatial memory deficits were dose-dependently alleviated by concomitant GR3027 treatment.

#### 3.2.4. GR3027 and AP Effects on Swimming Behavior 

The swim speed of the group treated with GR3027 alone was lower (F (6, 82) = 3.244; *p* = 0.007) than in the groups treated with AP or AP:GR3027 combinations (*p* < 0.05) but no different compared to the vehicle group (*p* > 0.05). No other differences in swim speed were found between the treatment groups and the swim speed was unchanged over time in all groups. The level of floating was, in general, low; it was unchanged over time and no statistical differences in floating were found between the groups. These findings suggest that the swim speed and behavior of floating were not especially affected by AP, GR3027, or combined AP and GR3027 treatment.

#### 3.2.5. GR3027 and AP Effects on Swim Pattern

**Figure 7:**
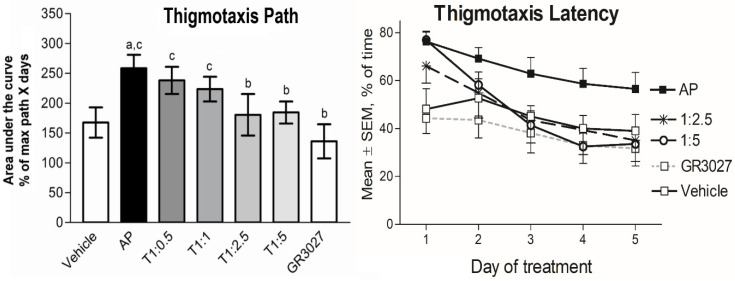
The AP-induced thigmotaxis behavior was attenuated by GR3027 in the MWM trials. Data are shown as mean area under the curve ± SEM for thigmotaxis path (**left** picture), length of swim spent close to the wall (% path of max path x days). On the (**right** picture) is % of time (max. 120 s) spent close to the wall in the Morris water maze for the treatment groups during the five days of testing. Treatments were vehicle (10% Captisol; n = 6), AP (2.2 mg/kg in β-CD; n = 20), T1:0.5 (AP:GR3027; 2.2:1.1 mg/kg in β-CD; n = 7), T1:1 (2.2:2.2 mg/kg in β-CD; n = 20), T1:2.5 (2.2:5.5 mg/kg in β-CD; n = 10), T1:5 (2.2:11 mg/kg in β-CD; n = 15), and GR3027 (11 mg/kg in β-CD; n = 11). Daily i.v. injections were administered 8 min before each rat’s first swim of the day. Letters above bars in left picture indicate statistically significant difference. a = vehicle compared to AP, T1:0.5, T1:1, b = AP compared to T1:2.5, T1:5, GR3027, c = GR3027 compared to AP, T1:0.5, T1:1, and d = T1:5 compared to T1:0.5, T1:1 (see Table 3 for *p*-values).

AP treatment changed the ways that the rats behaved in the pool in the sense that a larger fraction of the time and path was spent swimming close to the pool wall (thigmotaxis). All groups but the vehicle group showed significantly less thigmotaxis over time (both % thigmotaxis of time: Table 3A and that of path: Table 3B, Figure 7), and all groups but the vehicle and the GR3027 alone started at high levels of thigmotaxis, which declined over time (individual groups’ repeated-measures analyses showed *p* < 0.05). The group given AP treatment showed a higher level of thigmotaxis (of time: Table 3A; Figure 7 left, and that of path: Table 3B; Figure 7 right) compared to the group given vehicle treatment; however, it was significant for thigmotaxis of the path only. The level of thigmotaxis in the GR3027 group did not differ compared to the vehicle group, and it was significantly lower than in the AP group. The level of thigmotaxis (AUC) in the groups given the T1:5 and T1:2.5 treatments, respectively, was significantly lower than in the group given AP alone and no different compared to the vehicle or GR3027 groups, respectively. The groups given T1:1 and T1:0.5, respectively, showed no differences in the level of thigmotaxis compared to the group given AP and significantly more thigmotaxis than the groups given the vehicle and GR3027, respectively. These data suggest that AP induced a swim pattern with thigmotaxis behavior, which was attenuated by simultaneous GR3027 treatment. 

#### 3.2.6. Pharmacokinetic Analysis of GR3027

Blood/plasma samples were obtained at several time points after the i.v. and p.o. administration of GR3027 to rats. The maximum plasma concentration (Cmax) and the time to Cmax (Tmax) were derived directly from the plasma concentration data. The results are shown in Table 4 as the area under the concentration–time curve (AUC, 0–24 h), elimination half-life in plasma (t_1/2_), elimination rate constant (Ke), total body clearance (CL), and mean residence time (MRT), i.e., how long GR3027 remained in the body. The tentative oral bioavailability (F) was calculated by dividing the dose-normalized AUC after p.o. administration by the dose-normalized AUC after i.v. administration, i.e., F = [(AUC(p.o.)/Dose(p.o.))///(AUC(i.v)/Dose (i.v.))], and reported as percentages (%). 

## 4. Discussion

In this study, we investigated the modulatory properties of GR3027 regarding AP-induced spatial memory deficits in rats using the MWM. Firstly, we replicated previously reported data confirming that AP induces spatial memory deficits [20,21]. We also showed that GR3027, on its own, has no effect on memory in the MWM. However, GR3027 significantly alleviated the AP-induced learning deficit in a dose-dependent manner. Effects of GR3027 on AP-induced memory deficits in normal rats have not been reported previously. GR3027 has, however, been evaluated in humans, namely in patients with hepatic encephalopathy. In this patient group, GR3027 improves EEG, vigilance, and cognition, supporting the findings in this study [23].

The learning was not completely impaired by the dosage of the AP treatment. Although significantly impaired, the rats in the present study did display an improvement in finding the platform. This shows that the dosage of AP was low enough to impair learning but did not cause anesthesia or the total inhibition of learning. This was also shown by the absence of an effect on swim speed. This is important as we wished to be able to detect both alleviation and deterioration in learning by the combined GR3027 + AP treatment.

While we observed low levels of floating in general and identified no changes in the floating pattern, we did observe a change in swim speed. After treatment with GR3027 alone, the rats had slower swim speeds than those in the AP alone and AP combination treatments (Figure 6). The reason for this is unclear and it may be an artifact, as the swim speed of the non-treatment group differed compared to that of the vehicle group. Nonetheless, alterations in swim speed may affect the interpretation of the results on latency, as a decreased swim speed leads to longer latency in finding the platform. Therefore, importantly, nearly identical results were found in the path to find the platform. Since the path was not directly affected by the swim speed, we conclude that the finding for the swim speed is of minor relevance.

As shown before, the swim pattern was changed by AP [20], as the thigmotaxis, or the % of time or path spent swimming close to the pool wall, was increased. Moreover, this change induced by AP was antagonized by GR3027 (ratio 1:2.5). The reason for the increase in thigmotaxis caused by AP is not known. Thigmotaxis in the water maze has sometimes been interpreted as a sign of anxiety, i.e., a way to avoid the frightening open area while swimming out from the wall. However, AP is usually considered anxiolytic, as with other positive GABA_A_ receptor modulators, but is shown to be anxiogenic in certain situations [39].

We also investigated the effect on anesthesia in this study, measured as the loss of the righting reflex. AP was given i.v. dissolved in cyclodextrin solution. Moreover, in these studies, GR3027 showed an antagonistic effect towards AP in a ratio of 1:7.5. The amount of GR3027 needed to antagonize was higher than for thigmotaxis and spatial memory. This may be due to the receptor subtypes engaged in anesthesia (α1) compared to memory (α5) [40]. There is also another factor: the volume of cyclodextrin needed to inject a dose of 15 mg/kg is quite large, and we know that large volumes of cyclodextrin can reduce the efficiency of the compound.

We investigated in vitro the antagonistic properties of GR3027 regarding the THDOC-dependent potentiation of GABA, primarily on extra-synaptic GABA_A_ receptors consisting of recombinant human α5β3γ2 subunits expressed in HEK cells, using the patch clamp technique. It has been previously shown that certain neurosteroids, including AP and THDOC, may modulate and directly activate GABA_A_ receptors, i.e., they may act both in the presence and the absence of GABA. Therefore, we first verified the effect of THDOC on the baseline holding current without the agonist-induced activation of GABA_A_ receptors. The presented results demonstrate that, also in our setting, THDOC may directly activate α5β3γ2 GABA_A_ receptors expressed in HEK-293 cells and positively modulate their responses to low concentrations of GABA, which was previously shown for GABA_A_ receptors studied in neurons and HEK-293 cells [36,41,42]. The aims of these studies were to find out whether GR3027 inhibits the effect of the positive allosteric modulator THDOC with and without GABA under physiological conditions. In addition, we wished to investigate whether GR3027 inhibited GABA’s effect. Using a protocol designed to mimic extra-synaptic “tonic inhibition” conditions with the long-term application of a low GABA concentration, we studied whether GR3027 could modulate the THDOC-mediated potentiation. Our findings revealed that GR3027 could antagonize both the direct THDOC-mediated gating effect of α5β3γ2L GABA_A_ receptors and the THDOC-mediated potentiation of the GABA-evoked activation of α5β3γ2L GABA_A_ receptors, without its own direct gating effect on the GABA_A_ channel function, within the tested concentration range, and this confirms earlier reports [35].

The α1 receptor subunit is generally regarded as an “intra-synaptic all-round” subunit [4,5,40]. The results suggest that both types of GABA_A_ receptors tested under physiological conditions in this study were affected by GR3027. The reason for using THDOC instead of AP in electrophysiological recordings is that THDOC has a faster washout rate and is thus easier to wash off and more suitable to use in vitro [8]. It has been shown that AP and THDOC bind to the same binding sites on the GABA_A_ receptor, both for the modulation of the GABA effect and for their own agonistic activity [7]. Furthermore, we have previously shown that the effect of AP and THDOC on chloride ion influx is similar [43]. We interpret that the current electrophysiological findings obtained using THDOC are highly relevant for interpretations of GR3027′s effects on AP-induced learning deficits. To be able to compare AP and THDOC’s dose relations in vitro, we investigated the effect of low-dosage AP on the current response in dissociated hypothalamic cells. We showed that AP and THDOC had effects in the same dose range in hypothalamic cells taken from male rats and on recombinant HEC cells.

The α5 receptor subunit is highly involved in learning and memory processes. Increased activation of the α5-containing receptors is believed to hinder learning and memory. A mouse model lacking the α5 subunit displayed enhanced learning [17]. Blockade of the GABA_A_ receptor subunit α5 increased learning and memory [18]. In addition, a specific α5 inverse agonist on the GABA_A_ receptor was shown to improve memory and learning in rodents [19]. In this paper, we show that GR3027 is an active GABA_A_ receptor-modulating steroid antagonist on α5-containing receptors and it is therefore likely that GR3027 antagonized the AP-induced learning deficits by antagonizing the AP-mediated modulation of the GABA response on the α5-containing receptors. However, GR3027′s antagonizing effect on the AP-induced loss of righting reflex demanded a higher AP:GR3027 ratio than the effect on learning and memory.

In conclusion, we have shown that GR3027 antagonized the AP-induced deficit and the THDOC-induced current increase for both α1-containing and α5-containing GABA_A_ receptors. GR3027 can also antagonize AP-induced memory and learning impairments. This would imply that the compound is effective for the alleviation of AP-induced learning deficits.

## Figures and Tables

**Table 1:** Treatment regime for animals in the MWM trial and the pharmacokinetic study, respectively.

Treatment	Vehicle	AP (mg/kg)	GR3027 (mg/kg)	Solution Mix (AP:GR3027) *	*n*	Adm.	Test
AP alone	β-CD	2.2	0	N/A	20	IV	MWM
GR3027	β-CD	0	11	N/A	11	IV	MWM
T1:0.5	β-CD	2.2	1.1	1.33:0.67	7	IV	MWM
T1:1	β-CD	2.2	2.2	1:1	20	IV	MWM
T1:2.5	β-CD	2.2	5.5	0.57:1.43	10	IV	MWM
T1:5	β-CD	2.2	11	0.33:1.67	15	IV	MWM
Vehicle	β-CD	0	0	N/A	6	IV	MWM

AP—allopregnanolone, β-CD—2-hydroxypropyl-β-cyclodextrin, IV—intravenous injection, MWM—Morris water maze, * AP and GR3027, respectively, were dissolved in 10% β-CD using ultrasound to concentrations of 2.0 mg/mL and the solutions were then mixed to yield the given proportions.

**Table 2:** Results from statistical analyses of learning performance in the MWM trials.

A. Latency to Find the Platform				B. Path to Find the Platform			
2-Way ANOVA Within-Subjects Effect				2-Way ANOVA Within-Subjects Effect			
Training day	F (4, 328) = 460.065		*p* < 0.001	Training day	F (4, 328) = 40.172		*p* < 0.001
Interaction with treatment	F (24, 328) = 1.967		*p* = 0.005	Interaction with treatment	F (24, 328) = 1.993		*p* = 0.004
1-Way ANOVA (Area under the Curve)				1-Way ANOVA (Area under the Curve)			
Treatment (T)	F (6, 82) = 40.063		*p* = 0.001	Treatment (T)	F (6, 82) = 6.648		*p* < 0.001
Post Hoc (Area under the Curve)				Post Hoc (Area under the Curve)			
Treatment	vs. Vehicle	vs. AP	vs. GR3027	Treatment	vs. Vehicle	vs. AP	vs. GR3027
Vehicle	-	*p* = 0.003	*p* = 0.924	Vehicle	-	*p* = 0.002	*p* = 0.551
AP	*p* = 0.003	-	*p* < 0.001	AP	*p* = 0.002	-	*p* < 0.001
T1:0.5 *	*p* = 0.013	*p* = 0.950	*p* = 0.006	T1:0.5 *	*p* = 0.005	*p* = 0.859	*p* < 0.001
T1:1 *	*p* = 0.014	*p* = 0.400	*p* = 0.004	T1:1 *	*p* = 0.002	*p* = 0.964	*p* < 0.001
T1:2.5 *	*p* = 0.213	*p* = 0.044	*p* = 0.174	T1:2.5 *	*p* = 0.143	*p* = 0.054	*p* = 0.017
T1:5 *	*p* = 0.259	*p* = 0.011	*p* = 0.211	T1:5 *	*p* = 0.177	*p* = 0.014	*p* = 0.018
GR3027	*p* = 0.924	*p* < 0.001	-	GR3027	*p* = 0.551	*p* < 0.001	-

* Combined treatment with dose ratio given as AP:GR3027.

**Table 3:** Results from statistical analyses of thigmotaxis behavior in the MWM trials.

A. % Thigmotaxis of Time				B. % Thigmotaxis of Path			
2-Way ANOVA Within-Subjects Effect				2-Way ANOVA Within-Subjects Effect			
Training day	F (3.0, 245.3) = 51.414	*p* < 0.001		Training day	F (3.0, 246.9) = 50.165		*p* < 0.001
Interaction with treatment	F (17.9, 245.3) = 2.440	*p* = 0.001		Interaction with treatment	F (18.1, 246.9) = 2.576		*p* < 0.001
1-Way ANOVA (Area under the Curve)				1-Way ANOVA (Area under the Curve)			
Treatment	F (6, 82) = 2.511	*p* = 0.028		Treatment	F (6, 82) = 2.973		*p* = 0.011
Post Hoc (Area under the Curve)				Post Hoc (Area under the Curve)			
Treatment	vs. Vehicle	vs. AP	vs. GR3027	Treatment	vs. Vehicle	vs. AP	vs. GR3027
Vehicle	-	*p* = 0.061	*p* = 0.512	Vehicle	-	*p* = 0.033	*p* = 0.492
AP	*p* = 0.061	-	*p* = 0.002	AP	*p* = 0.033	-	*p* < 0.001
T1:0.5 *	*p* = 0.189	*p* = 0.741	*p* = 0.029	T1:0.5 *	*p* = 0.164	*p* = 0.603	*p* = 0.022
T1:1 *	*p* = 0.270	*p* = 0.251	*p* = 0.026	T1:1 *	*p* = 0.186	*p* = 0.222	*p* = 0.011
T1:2.5 *	*p* = 0.875	*p* = 0.042	*p* = 0.344	T1:2.5 *	*p* = 0.782	*p* = 0.028	*p* = 0.262
T1:5 *	*p* = 0.885	*p* = 0.020	*p* = 0.311	T1:5 *	*p* = 0.701	*p* = 0.018	*p* = 0.181
GR3027	*p* = 0.512	*p* = 0.002	-	GR3027	*p* = 0.492	*p* < 0.001	-

* Combined treatment with dose ratio given as AP:GR3027.

**Table 4:** Mean pharmacokinetic parameters of GR3027 based on plasma concentrations after p.o. and i.v. administrations. The values are calculated from actual accurate time points.

Parameter (Mean)	2 mg/kg i.v.	10 mg/kg p.o.
AUC (min ∗ ng/mL)	2205586	113568
Cmax (ng/mL)	74506	504
Tmax (min)	5	34
t½ (min)	677	310
Cl (ml/min/kg)	1.09	-
MRT (min)	16.6	284
Ke (1/min)	0.0012	0.0025
F(%)	-	1.0

## Data Availability

The data are owned by a biotech company and are not available due to business secrecy.
